# One-to-one counselling and school attendance in the UK: a single group pre-post study

**DOI:** 10.1136/archdischild-2023-326458

**Published:** 2024-07-29

**Authors:** Jennifer Saxton, Katalin Toth, Obioha C Ukoumunne, Hannah Wilkinson, Jemma White, Sarah Golden, Tamsin Ford

**Affiliations:** 1 Psychiatry, University of Cambridge, Cambridge, UK; 2 Place2Be, London, UK; 3 NIHR CLAHRC South West Peninsula, University of Exeter Medical School, Exeter, UK

**Keywords:** Mental health, Adolescent Health, Child Health, Child Psychiatry, Child Health Services

## Abstract

**Objective:**

Absence rates remain high in UK schools, with negative implications for attainment, life chances and inequality. Reasons for non-attendance are complex but include psychosocial factors. Few UK-based studies have evaluated psychosocial interventions for school attendance outcomes or its moderators. This pre-post evaluation examined the potential influence of school-based one-to-one counselling on school attendance and possible moderators.

**Design and setting:**

Secondary analysis of routine data, collected by a national mental health provider in primary and secondary schools.

**Participants:**

7405 pupils aged 4–19 years, with complete school attendance records at Time1 (pre-counselling term) and Time2 (the term when counselling ended).

**Intervention:**

All participants received school-based one-to-one counselling with a trained counsellor between August 2016 and December 2019.

**Outcomes:**

Percentage of school sessions attended (continuous) and persistent absence (binary; attending ≤90% of sessions) in a term. Potential moderators included sociodemographics, mental health and school engagement/enjoyment.

**Results:**

Median Time1 attendance was 96%. 23.6% of participants were persistently absent. The intervention was not associated with improved percentage attendance (0.028%, 95% CI −0.160–0.216%) but was associated with 18.5% reduced odds of persistent absence (OR=0.815, 95% CI 0.729–0.911). We identified five moderators of change in attendance (interaction terms p<0.05): age group (improvements for 4–9 s; worsening for 15–19 s), improvement for some ethnicities and lower parent/carer education. Mental health and school engagement/enjoyment co-varied with attendance in expected directions.

**Conclusions:**

One-to-one counselling may improve school attendance among persistently absent pupils, particularly at younger ages. Improving mental health and pupil engagement/enjoyment are potential intervention targets. Our hypotheses require confirmation with controlled designs.

**WHAT IS ALREADY KNOWN ON THIS TOPIC**

**WHAT THIS STUDY ADDS**

- Few UK-based interventions have included school attendance outcomes.
- We examined the relationship between UK school-based one-to-one counselling and school attendance.

**HOW THIS STUDY MIGHT AFFECT RESEARCH, PRACTICE OR POLICY**

- One-to-one counselling may reduce persistent absence in younger children.
- Promising intervention targets include mental health and pupil engagement/enjoyment.

## Introduction

### Background

 School attendance is positively associated with educational attainment^1 2^ and can boost children’s life satisfaction and happiness.^3^ Despite these benefits, school absence rates (the proportion of school sessions missed) in England were 4.7–4.8% in the three years before the COVID-19 pandemic and stand at 7.4% (2022/2023).^4^ Absence rates vary by pupil characteristics, for example, they are higher for secondary school children and those receiving free school meals (FSMs).^4^ Persistent absence (≤90% of school sessions attended)^5^ almost doubled between 2018/2019 and 2022/2023 (from 10.9% to 21.2%)^4^ and is the focus of a recent Education Committee enquiry.^6^

Reasons for school non-attendance are multifaceted. Risk factors include depression, anxiety,^7^ psychotic disorders, conduct problems,^8^ pupil disengagement,^9^ school dissatisfaction,^10^ poor parent/carer mental health^11^ and neurodiversity.^8^ Socioeconomic disadvantages are independently associated with poorer attendance^12^ and contribute to attainment inequalities.^13^ The Children’s Commissioner’s attendance audit highlighted bullying and problems at home as additional attendance barriers.^14^ The audit suggested that schools provide early support services, such as school-based counselling, which is pertinent given increasing rates of probable mental disorder among children in England.^15^ Government policy is to strengthen prevention and early treatment of mental health problems via school-based Mental Health Support Teams, but their impact on school attendance is unknown.^16^

### Rationale

The small evidence-base for school attendance interventions is dominated by USA-based studies,^17^ suggests that drivers of poor mental health and pupil engagement are meaningful targets,^18^ and responsive individually tailored approaches show promise.^19^ Shortcomings include limited sociodemographic and moderator-based analyses^7 20^ and few UK-based studies.^19^ Background disadvantage drives attendance inequalities, so equity of outcomes are important considerations. To our knowledge, there are no UK school-based evaluations of mental health interventions to improve overall school attendance.

### Aims

This study aimed to test whether UK school-based one-to-one counselling is associated with improved school attendance.

### Objectives

1. Quantify associations between one-to-one counselling and school attendance (percentage of school sessions attended and persistent absence in a given term).
2. Identify moderators of the association between one-to-counselling and change in school attendance (eg, age, FSM status).
3. Assess whether changes in mental health and school engagement/enjoyment are associated with changes in school attendance.

## Methods

### Design, setting, participants

Our single group, pre-post comparison design used routine monitoring data collected by a national organisation supporting children’s mental health in schools (Place2Be). The intervention was delivered in 392 primary and secondary schools across England, Scotland and Wales. Data represent an open cohort of children whose one-to-one counselling episodes (which varied in number of sessions) began (Time1) and ended (Time2) anytime between August 2016 and December 2019. Counselling episodes were defined by the dates of a child’s first and last counselling session.

Participants were aged 4–19 years, referred (self, teacher or parent/carer) for one-to-one counselling for an identified mental health need requiring full assessment. Cases were included provided the pupil attended primary or secondary school and had complete school attendance data at Time1 (the pre-counselling term) and Time2 (the term counselling ended) and had equal to or more than one counselling session.

### Intervention

School-based one-to-one counselling was delivered by trained counsellors (Level-4 UK qualifications framework) after initial case formulation.^21^ Anecdotally, there are high levels of intervention offer and uptake after assessment, as staff are embedded within schools, allowing for prior discussion with young people, teachers and/or parents/carers. Counselling sessions were weekly, lasting 40–60 min. The total sessions offered varied by need (mean=24.9 sessions, SD=13.4, median=24, IQR=14–33, minimum=1, maximum=127). Parents/carers were offered partnership meetings with counsellors alongside children’s counselling, of which 71% attended at least one (see online supplemental material).

### Outcomes

School attendance was recorded at Time1 and Time2 in the case information database, comprising routine data compiled by counsellors, collected from parents/carers, young people, teachers (independent self-report or verbally depending on respondent preference) and school records.

1. Primary outcome: Percentage school attendance (hereafter SA, continuous variable)—The percentage of school sessions attended in the term, out of the total sessions offered.
2. Secondary outcome: Persistent absence (hereafter PA, binary variable)—The percentage of pupils attending ≤90% of the total sessions offered in the term.

### Potential moderators and descriptive variables

Variables drawn from the case information database included sociodemographics, parent/carer mental health, special educational needs (SEN) status, school type, child mental health (Strengths and Difficulties Questionnaire (SDQ)^22^ to estimate probable mental disorder status and change over time), school engagement/enjoyment (six teacher-rated items generating total scores and change over time, measured in primary school pupils only) and intervention-related variables (eg, counselling duration). See online supplemental material for details of variables, their sources and recoding.

### Statistical methods

All statistical analyses were performed in Stata-SE, V.17.

### Descriptive statistics

Continuous variables are summarised using means and SD or medians and IQR, and categorical variables are summarised using numbers and percentages.

### Main and moderator associations

We fitted mixed-effects regression models to compare outcomes between Time1 and Time2. Models were fitted to repeated measures data so outcome data from both time points were analysed as a single variable, where ‘time’ was the main predictor to represent the effect of the intervention. The models allowed for the nested data structure: observations within pupils, within schools, within local authorities.^23^

We used tests of interaction to assess whether the association between ‘time’ and the outcome differed across categories defined by potential moderators. If interactions were statistically significant (p<0.05), we used Stata’s margins command to obtain estimated mean SA at Time1 and Time2 for each level of the moderator.

### Missing data

We report proportions and treatment of missing data for all variables in tables1  2 and online supplemental material.

**Table 1:** Time1 characteristics and school attendance of children who received one-to-one counselling (n=7405)*

Participant characteristic	Total n within sample (%)	Median (IQR) % school attendance the term before counselling	N (%) Persistent absence† the term before counselling
Child age (years)			
4–9	4181 (56.5)	96 (92–99)	887 (21.2)
10–14	2996 (40.5)	95 (90–99)	779 (26.0)
15–19	228 (3.1)	94 (86–98)	83 (36.4)
Gender			
Male	3921 (53.0)	96 (91–99)	869 (22.2)
Female	3484 (47.1)	96 (90–99)	880 (25.3)
Ethnicity			
White British	4199 (56.7)	95 (90–99)	1077 (25.7)
White Irish/other	495 (6.7)	96 (91–99)	115 (23.2)
Asian/Asian British/Chinese	593 (8.0)	97 (92–99)	122 (20.6)
Black/black British	974 (13.2)	97 (92,99)	181 (18.6)
Mixed ethnicity	737 (10.0)	96 (91–99)	169 (22.9)
Any other ethnic group/ preferred not to say/missing	407 (5.5)	96 (92–99)	85 (20.9)
Parents’ highest education level			
No qualification	782 (10.6)	94 (89–98)	244 (31.2)
GCSEs at grades D–G or equivalent	497 (6.7)	95 (90–98)	134 (27.0)
GCSEs at grades A*–C or equivalent	694 (9.4)	96 (91–99)	164 (23.6)
A levels/highers or equivalent	678 (9.2)	97 (93–100)	130 (19.2)
≥Degree, level 4 NVQ/SVQ	1005 (13.6)	97 (93–99)	168 (16.7)
Missing or unknown	3749 (50.6)	96 (91–99)	909 (24.3)
Parent/carer mental health difficulties			
Never experienced	3814 (51.5)	96 (92–99)	769 (20.2)
Currently or within 6 months	1367 (18.5)	95 (89–99)	400 (29.3)
At least 6 months ago	679 (9.2)	95 (90–98)	183 (27.0)
Preferred not to say or missing	1545 (20.9)	96 (90–99)	397 (25.7)
Receiving pupil premium			
Yes	3353 (45.3)	95 (90–99)	883 (26.3)
No	3374 (45.6)	97 (92–99)	643 (19.1)
Unknown	678 (9.2)	95 (88–98)	223 (32.9)
Receiving free school meals			
Yes	2598 (35.1)	95 (89–98)	745 (28.7)
No	3238 (43.7)	96.5 (92–99)	661 (20.4)
Missing/unknown	1569 (21.2)	96 (91–99)	343 (21.9)
Special educational needs (SEN)			
No support provided	5281 (71.3)	96 (91–99)	1203 (22.8)
SEN support or equivalent‡	1857 (25.1)	96 (90,99)	483 (26.0)
EHCP or equivalent§	267 (3.6)	96 (91–98)	63 (23.6)
School stage of child			
Primary school	5891 (79.6)	96 (92–99)	1233 (20.9)
Secondary school	1514 (20.5)	94 (88–98)	516 (34.1)
School type¶			
Academies and free schools	2854 (38.5)	96 (91–99)	705 (24.7)
Community schools**	4429 (59.8)	96 (91–99)	1020 (23.0)
Other independent schools	122 (1.7)	96 (92–100)	24 (19.7)
**Total**	**7405** (**100**)	**96** (**91–99**)	**1749** (**23**.**6**)

* The denominator includes data from first counselling episodes only if young people had received >1 counselling episode.

† Persistent absence: ≤90% school attendance of total offered school sessions/term, see Department for Education’s 2019 definition (https://assets.publishing.service.gov.uk/government/uploads/system/uploads/attachment_data/file/468924/Guide_to_absence_statistics_15102015.pdf).

‡ ‘SEN support’ is considered equivalent to ‘school action’/’school action plus’ (Wales) and ‘additional support needs’ (Scotland).

§ EHCP=Education and Health Care Plan, considered equivalent to ‘full statement’ (Wales) and ‘coordinated action plan’ (Scotland).

¶ School types originated from the Department for Education website, and were recoded into a smaller number of categories (https://www.gov.uk/types-of-school, date accessed 05/07/2022).

** Community schools include community schools, community special schools, foundation schools, voluntary controlled schools, voluntary aided schools, all schools from Scotland and Wales (neither shares England’s academy system).

**Table 2:** Estimated main effect and moderator associations between one-to-one counselling and percentage school attendance (outcome 1) and persistent absence (0=no, 1=yes; outcome 2)

Intervention (main effects)*		% school attendance†		Persistent absence‡	
		Mean change	95% CI	OR	95% CI
First episode of counselling		0.028	−0.160, 0.216	0.815	0.729, 0.911
Main effect: sensitivity analyses§		0.024	−0.165, 0.212	0.829	0.741, 0.926
Moderator marginal effects (ref: Time1)¶					
Ethnicity##time	White British	−0.127	−0.377, 0.122	0.985	0.974, 0.995
	White Irish/other	0.200	−0.530, 0.927	0.991	0.961,1.022
	Asian/Asian British/Chinese	0.614	0.050, 1.278	0.952	0.920, 0.981
	Black/black British	0.204	−0.314, 0.723	0.991	0.967, 1.015
	Mixed ethnicity	−0.076	−0.672, 0.520	0.999	0.974, 1.024
	Any other/preferred not to say/missing	0.339	−0.463, 1.141	0.989	0.955, 1.024
Age group##time	4–9 years	0.276	0.026, 0.526	0.978	0.967, 0.990
	10–14 years	−0.185	−0.480, 0.111	0.988	0.975, 1.000
	15–19 years	−1.719	−2.79, 0.649	1.064	1.005, 1.124
Parent/carer highest education##time	No qualifications	N/A	N/A	0.958	0.926, 0.989
	L1, GCSEs at grades D–G or equivalent	N/A	N/A	0.978	0.940, 1.016
	L2, GCSEs at grades A*–C or equivalent	N/A	N/A	0.971	0.940, 1.002
	L3, A levels/highers or equivalent	N/A	N/A	0.985	0.955, 1.015
	L4, degree, level 4 NVQ/SVQ or above	N/A	N/A	0.956	0.934, 0.979
	Missing or unknown	N/A	N/A	0.998	0.984, 1.011
Free school meals status##time	Not receiving FSM	−0.237	−0.521, 0.047	N/A	N/A
	Receiving FSM	0.061	−0.256, 0.378	N/A	N/A
	FSM status missing/unknown	0.522	0.114, 0.930	N/A	N/A
School stage##time	Primary school	0.287	0.077, 0.497	0.979	0.970, 0.989
	Secondary school	−0.978	−1.393, to 0.563	1.001	0.981, 1.021
Planned ending to counselling	No	−1.305	−1.638, to 0.971	1.016	1.001, 1.032
	Yes	0.640	0.414, 0.865	0.968	0.957, 0.978
SDQ status (‘probable disorder’) change between times 1 and 2**##time	Stays in unlikely	1.040	0.473, 1.598	0.931	0.902, 0.960
	Deteriorates	0.332	−0.398, 1.061	0.987	0.949, 1.024
	Improves	0.846	0.586, 1.107	0.955	0.941, 0.968
	Stays in possible	0.484	−0.312, 1.280	0.977	0.936, 1.019
	Stays in probable	−0.830	−1.181, 0.479	1.021	1.003, 1.039
Engagement and enjoyment (EE) at school score (continuous) change between times 1 and 2††##time	EE score difference: −14	−1.874	−2.782, 0.965	1.114	1.058, 1.170
	EE score difference: −9	−1.108	−1.739, 0.478	1.070	1.032, 1.107
	EE score difference: −4	−0.343	−0.714, 0.029	1.025	1.003, 1.047
	EE score difference: 1	0.423	0.203, 0.643	0.980	0.967, 0.993
	EE score difference: 6	1.189	0.832, 1.546	0.936	0.914, 0.957
	EE score difference: 11	1.954	1.341, 2.567	0.891	0.853, 0.928
	EE score difference: 16	2.720	1.829, 3.610	0.846	0.790, 0.901

* Main effects tested with separate mixed effects linear and logistic regression models including time (intervention) and outcome variables, accounting for clustering at individual, school and local authority levels.

† % school attendance=% of school sessions attended/offered by each school; generally two possible sessions per day (morning and afternoon).

‡ Persistent absence (0=no, 1=yes) is defined by the Department for Education as ≤90% school attendance out of all possible school sessions offered.

§ Sensitivity analyses: first episode of counselling replaced with last episode for those with more than one counselling episode in the dataset.

¶ Moderators tested in separate models were: gender, ethnicity, age group, parent/carer highest education level, free school meals status, pupil premium status, parent/carer mental health status, SEN status, primary versus secondary school pupil, planned ending to counselling (yes/no), SDQ status change over time, engagement and enjoyment of school score change over time. Only moderators with interaction terms significant at p<0.05 for at least one subgroup were further examined using Stata’s margins commands and presented in the table for all subgroups for a given variable. For simplicity, we have only presented moderators with significant interaction terms in this table.

** SDQ=Strengths and Difficulties Questionnaire. Single probable disorder scores were calculated based on the SDQ predictive algorithm code available here, which incorporates up to 12 input variables from three respondents (child, teacher, parent/carer) about hyperactivity, conduct, emotional and total impact scores: https://www.sdqinfo.org/c4.html (accessed August 2022). Self-reported SDQ data were not collected from children under 11 years of age, so for children 4–10 years of age, the algorithm was based on two respondents only: parent/carer and teacher. SDQ data were missing on ≥1item for n=924/7405 of the sample.

†† Time2-Time1 difference, difference scores ranged from -14 to +16 and we used Stata’s margins dydx command to estimate mean slopes at several cut-points along the range of possible scores. Negative scores indicate worsened enjoyment, 0 no change, positive scores show improvement); teacher reported items for a subset of the sample (3477/7405).

FSMfree school mealGCSEGeneral Certificate of Secondary EducationNVQNational Vocational QualificationSVQScottish Vocational Qualification

### Sensitivity analyses

A minority of children had two or three discrete counselling episodes recorded during the data collection period. In all analyses, we included only one counselling episode per child. In the main analyses, we prioritised children’s first counselling episodes (which for most children was their only episode). In sensitivity analyses, if multiple episodes were available for the same child, we used their last episode instead of their first to observe any changes to the findings and interpretation.

### Ethics

Parents/carers provided opt-in consent for deidentified data use for research, and the provider’s Research Advisory Group provided ethical oversight.

## Results

### Analytical sample

From 12 031 available records, we excluded 20 nursery-age children; two gender non-conforming children; 4421 children with school attendance recorded as missing or 0% at either time point. The remainder included 7225 children with one counselling episode, 177 children with two episodes and three children with three episodes, providing n=7405 first counselling episodes for analysis. χ^2^ tests with Cramer’s V to explore missing attendance data suggested minimal associations with sociodemographic variables (see online supplemental material).

### Intervention process information

Median (IQR) counselling duration was 7.4 months (4.1–10.2), ending by mutual agreement in 69% of cases. 83% of counselling sessions offered were attended; 5% were missed due to in or out-of-school absences.

### Participant characteristics

Median SA was high (96%), but 24% of children were PA.

Table 1 reports Time1 SA and PA by background characteristics. Time1 SA worsened with increasing age, white British ethnicity, lower parent/carer education, and current/recent parent/carer mental health problems. PA was higher among girls, children with SEN, pupil premium and FSM. Online supplemental material shows 68% of children had probable mental disorder at Time1 and worse Time1 SA than those unlikely to have disorders. PA was highest for children self-reporting severe pro-social difficulties. There was decreasing SA and increasing PA with decreasing Time1 school engagement/enjoyment.

### Intervention associations with school attendance

There was little evidence of change in SA at Time2 (mean change=0.03%, 95% CI=−0.16% to 0.22%), but the odds of PA were 18% lower (OR=0.82, 95% CI=0.73 to 0.91). Overall PA decreased from 23.6% to 21.9%. Findings were similar in sensitivity analyses.

Following tests of interaction (p<0.05), SA in the Asian/Asian British/Chinese group increased marginally (mean+0.6%) and odds of PA reduced by 0.9% at Time2; odds of PA reduced by 1.5% for children with white British ethnicity.

Mean SA increased by 0.3% at Time2 for children aged 4–9 years and reduced by 1.7% for those aged 15–19 years. There was a small decreased odds of PA among children aged 4–9 (2.2%) and 6.4% increased odds for those aged 15–19 at Time2. There were 4.4% and 0.2% reduced odds of PA if parents/carers had no educational qualifications and the highest qualifications, respectively.

SA in the FSM category ‘missing/unknown’ increased by a mean of 0.5% at Time2, although this category includes early years/reception up to year 2 for whom FSM are universal.

Mean SA for mutually concluded counselling episodes increased by 0.6% at Time2, with a 3.2% reduced odds of PA. Unplanned endings to counselling were associated with a 1.3% mean decrease in SA and 1.6% increased odds of PA.

**Figure 1:**
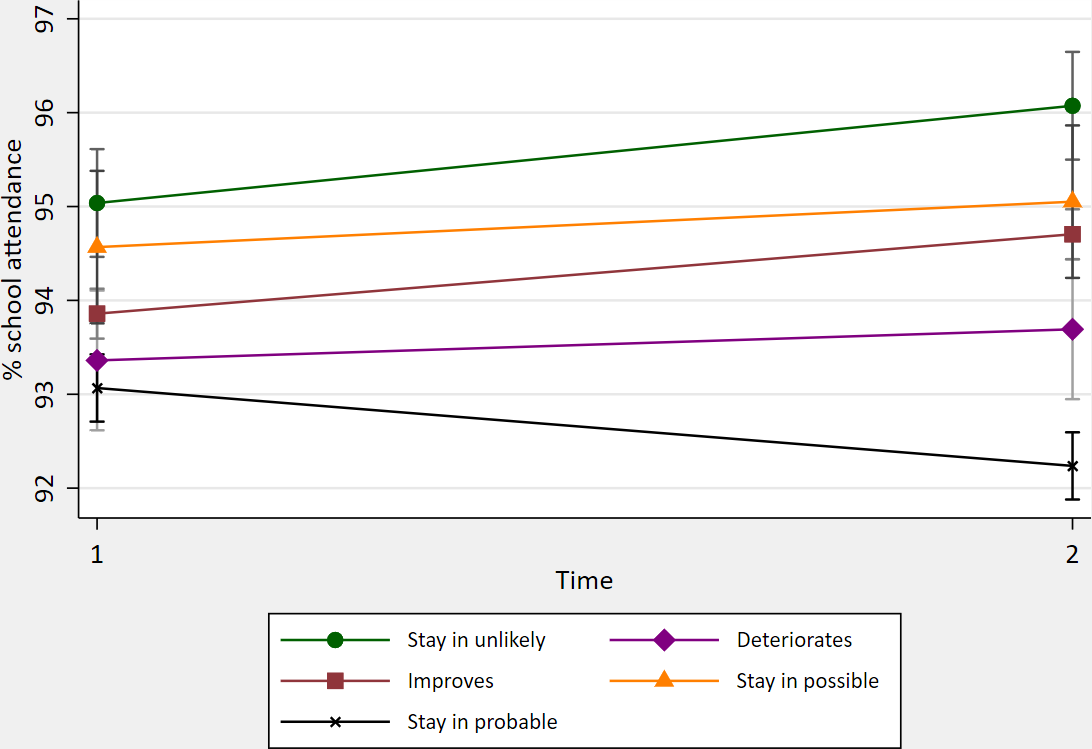
Adjusted mean % school attendance (95% CIs) at Time1 and Time2 by Strengths and Difficulties Questionnaire ‘probable disorder’ category.

Moderator interaction terms were not statistically significant (p≥0.05) for gender, parent/carer mental health, SEN and pupil premium status. The relationship between SDQ status and SA at Time1 and Time2 is shown in figure 1. Children remaining in the group ‘unlikely to have any disorder’, and those whose status improved, had improved mean SA over time (95.0–96.1% and 93.9–94.7%) and 6.9% and 4.5% reduced odds of PA. Mean SA of those remaining in the probable disorder group worsened (93.1–92.2%) and PA odds increased by 2.1% at Time2. Interactions were not significant for other SDQ groups. Examination of missing SDQ data suggests that our analysis includes children with better SA than the complete sample (see online supplemental material).

**Figure 2:**
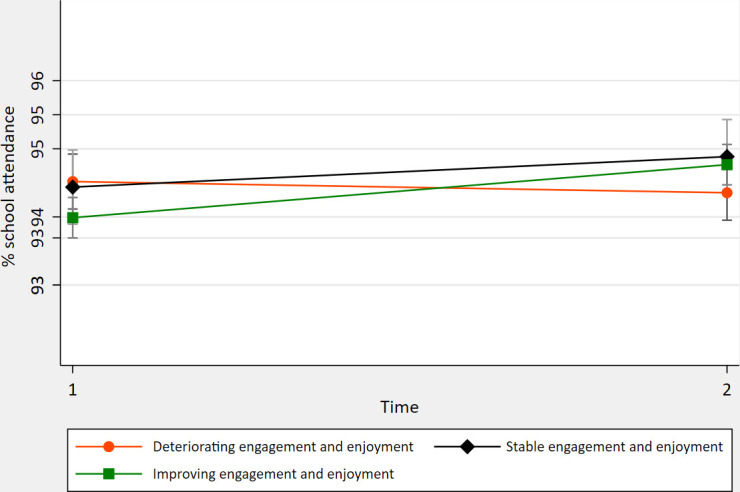
Adjusted mean % school attendance (95% CIs) at Time1 and Time2 by ‘school engagement and enjoyment’ category.

Figure 2 shows the relationship between change in school engagement/enjoyment over time (categorical variable) and SA. As a continuous variable (table 2), the greater the change in engagement/enjoyment, the greater the change in SA and PA.

## Discussion

We found no evidence that one-to-one counselling was associated with SA, but it was associated with 18.5% reduced odds of PA.

Sociodemographic moderators of the intervention-school attendance relationship included age group (positive for younger, negative for older) and were marginally positive for Asian/Asian British/Chinese children and if parents/carers had the lowest or highest educational qualifications. The moderating role of FSM was unclear. Children who completed their counselling episodes as planned had significantly better SA and lowered odds of PA at Time2, whereas attendance worsened if counselling ended unplanned.

Change in probable mental disorder status was associated with SA at Time2 (positively for children whose status improved or remained in the group ‘unlikely’ to have any disorder and negatively for children who deteriorated or remained in the ‘probable disorder’ group). Changes in school engagement/enjoyment coincided with changes in school attendance. The relationship appears strongest for those whose engagement/enjoyment increased the most.

### Strengths and limitations

This is the first UK-based study to assess the association between a school-based mental health intervention and overall school attendance based on a large sample of children and schools. Using in-depth routinely collected information from multiple respondents about the same child, we have identified potential moderators of the intervention-attendance relationship and generated hypotheses for testing with controlled designs.

Limitations include our single group pre-post design, so we cannot attribute our findings to the intervention. We also tested multiple interactions in simple models, which increase type I error risk and do not adjust for confounders. Similarly, there may be unmeasured and residual confounding.

Our convenience sample only included one special school, had substantially worse PA levels at Time1 compared with England-wide figures over the same period (23.6% vs 11.6–13.0%)^24^ and was more disadvantaged (eg, FSM was 15.4% in 2019 compared with 35.1% in our sample).^25^ Due to missing data, our SDQ moderator analysis included a subsample of children with better attendance than the overall sample. Our eligibility criteria required complete attendance data at both time points, so we excluded children who arrived partway through the Time1 term, or left the school during Time2, who may be more vulnerable. Finally, we could not differentiate authorised from unauthorised absences, absence reason or examine attendance patterns (eg, days of the week).

### Interpretation

Our evaluation detected no intervention association with SA, which could mean that measurable improvements are beyond the reach of one-to-one counselling. However, this null finding should be considered in the context of high Time1 median SA, and that SA included authorised absences. Our finding of reduced odds of PA suggests potential benefit for this subgroup but needs confirmation with a controlled design. There is limited comparable evidence from UK school-based settings, and PA definitions vary by country, but a meta-analysis of largely USA-based controlled studies targeting chronic absenteeism found small effect sizes for behavioural interventions and those supporting academic performance.^17^

Equity in attendance outcomes from psychosocial interventions has been highlighted as an evidence gap.^20^ We are encouraged that no equity-based subgroups were worse off at Time2 compared with Time1, and there was evidence that attendance improved among children whose parents/carers had no educational qualifications. Ethnicity could be explored further with a larger sample and fuller disaggregation. Our Time1 data align with other literature showing strong socially determined disparities in school attendance, including parent/carer education and mental health, and receiving FSM.^13^ Our finding that attendance worsened if counselling ended without mutual agreement could be further explored, by reason for cessation.

Improved outcomes for younger children could be explained by the greater influence of parents/carers on attendance at this age and because the intervention engaged parents/carers in designing mental health support for their children. The negative intervention-attendance association among adolescents matches age-related attendance patterns in government statistics.^4^

Our finding that changes in ‘probable disorder’ coincided with changes in school attendance aligns with evidence that mental health influences school attendance.^8^ Although we cannot conclude that the intervention improved mental health or be certain of temporal sequences, we hypothesise that mental health interventions offer an important pathway to improve school attendance. This is supported by effectiveness reviews of psychosocial interventions in other countries and settings.^18^

Changes in school engagement/enjoyment coincided with changes in attendance. USA-based studies report that student engagement predicts school drop-out among children with and without learning disabilities and emotional and behavioural disorders, adjusted for socioeconomic status and attainment,^9^ while middle-high school students with high school satisfaction had lower absenteeism.^10^ This suggests that promoting children’s engagement and enjoyment of school—within or outside of mental health interventions—may support school attendance.

### Future research recommendations

To build the UK evidence-base, one priority is to replicate and extend our findings using evaluation designs with controls. This will confirm whether one-to-one counselling is effective in improving school attendance and will provide a better understanding of equity-based and age-group effects, particularly for adolescents. Follow-up data could provide evidence for longer term benefits of intervening at younger ages, and linkage to referral data could help us understand if self-referrers were more motivated to complete the intervention. Testing our hypotheses in mediation and moderation analyses will provide further insight into the complexities underpinning school attendance, including bidirectional and temporal effects. Engagement/enjoyment could be further explored, with children to co-design new approaches promoting these factors within interventions and schools, to standardise the six-item scale we used, and in quantitative models to understand the extent engagement/enjoyment moderates and/or mediates the influence of mental health interventions on school attendance.

## Conclusion

One-to-one counselling has the potential to reduce persistent absence, particularly for younger children. Early mental health support and promoting positive educational experiences could improve school attendance and provide longer term benefits for children as they move into adolescence and young adulthood.

## supplementary material

10.1136/archdischild-2023-326458 online supplemental file 1

## Data Availability

Data are available upon reasonable request.
