# Supplementary material for: One-to-one counselling and school attendance in the UK: a single group pre-post study
Source: Arch Dis Child. 2024 Jul 29;109(11):e326458. doi: 10.1136/archdischild-2023-326458 (PMC11503188; doi:10.1136/archdischild-2023-326458)
Supplement: online supplemental file 1 [file archdischild-109-11-s001.pdf]

Supplemental material

Contents

Key messages 2

What is already known on this topic: 2

What this study adds: 2

How this study might affect research policy or practice 2

Author contributions statement: 3

Declaration of interests: 3

Data sharing statement: 3

Table S1: Summary data on the uptake of parent/carer partnership meetings for the period of the dataset (August 2016-December 2019, n=7588) 4

Overview of variables from case information database for analysis 5

Table S2: Descriptive and moderator variables for analysis 6

References to support Table S2 8

Table S3: Baseline mental health characteristics and presenting issues by baseline school attendance (n=7405[a] unless stated) 9

Chi-Square Tests and Cramer's V to explore missing outcome data 10

Table S4: Chi-Square and Cramer's V tests to assess bias in missing outcome data 10

The association of missing SDQ data with school attendance outcomes 11

Table S5: Teacher-reported items about engagement and enjoyment at school, sub-sample n=4678[a,b] 12

Key messages

What is already known on this topic: Most intervention research assessing school attendance outcomes originates from the USA, identifying small effect sizes for individualised approaches, parent engagement, academic support and specific psychosocial programmes. Gaps in the literature include a lack of focus on equity, little UK-based evidence to guide policy, and limited data about moderators of overall school attendance.

What this study adds: Our study is the first to evaluate a school-based mental health intervention and its moderators against overall school attendance outcomes in the UK, using a large sample of primary and secondary age children, with in-depth socio-demographic, mental health and pupil engagement data.

How this study might affect research policy or practice

Expanding the scale and remit of school-based mental health support, and strategies to improve pupil engagement and enjoyment could improve school attendance in the UK and decrease long-term harm and population inequality. Future interventions should consider differential age-group effects when designing content and process, equity of inclusion and outcomes, and include appropriate comparison groups.

Author contributions statement:

JS and KT directly accessed and verified the data in the underlying data reported in the manuscript. JS, TF, SG, KT, GW and OU contributed to the analysis plan. JS conducted the primary analysis and drafted the manuscript, tables and figures. All authors contributed to the interpretation of the data and critically revising the manuscript for important intellectual content, had full access to all the data in the study and accept responsibility to submit for publication.

Declaration of interests:

SG, KT, HW & JW declare that they are employed by the implementing organisation. There are no other conflicts of interest.

Data sharing statement:

The data collected for the study, including individual participant data and a data dictionary, will be made available to others on request. The data that will be made available includes de-identified participant data and a data dictionary. The following related documents will be available on request: statistical analysis plan and informed consent form.

The data will be available indefinitely from the data of publication. The data will be made available on request. It can be requested by email by contacting evaluation@place2be.org.uk.

Access to the data will be provided after approval of a proposal by the implementing organisation and a signed data access agreement. Users of the data will be required to share any published results with the implementing organisation.

Table S1: Summary data on the uptake of parent/carer partnership meetings for the period of the dataset (August 2016-December 2019, n=7588[a])

Number of partnership meetings dated August 2016-December 2019	Number of parents/ carers	Total % of parents/ carers
0	2200	29·0
1	1684	22·2
2	1542	20·3
3	947	12·5
4	529	7·0
5 or more	686	9·0
TOTAL	7588	100

a 7588 refers to the total number of counselling episodes recorded during the period covered by the dataset. Counselling episodes varied in duration and were defined using the dates of first and last counselling sessions. A minority of children had more than one counselling episode recorded over the timeframe August 2016-December 2019.

Overview of variables from case information database for analysis

1. Background characteristics: age, gender, ethnicity, parent/carer highest educational qualification, parent/carer mental health status, FSM status, Pupil Premium status, SEN status, primary or secondary school, school type;
2. Child mental health: 1) Strengths and Difficulties Questionnaire (SDQ)[1] to estimate probable mental disorder status and change over time; 2) Presenting issues to counselling;
3. School engagement/enjoyment: Six teacher-rated items, covering class concentration, effort and enjoyment, to generate total scores and change over time;
4. Intervention-related variables: counselling duration, attendance, and if episodes ended as planned.

Table S2: Descriptive and moderator variables for analysis

Variable name	Variable levels	Recode details	Data source(s)	Time periods collected
**Socio-demographic/background moderators**				
Child age (in years) at first counselling session	Age groups:<br>4-9<br>10-14<br>15-19	Age groups reflect early and middle childhood, and adolescence	Parents/carers of children <11 years, or young people (age 11+) via paper surveys, electronic questionnaire and/or via face-to-face meetings. A flexible approach is taken depending on respondent preference.	Time1 only
Gender	Male<br>Female<br>In another way	N/A - variable unchanged		
Ethnicity	1) White British 2) White Irish/Other 3) Asian/Asian British/Chinese 4) Black/Black British 5) Mixed Ethnicity 6) Any other ethnic group/preferred not to say/missing.	• Due to <5% cases available for analysis we combined 'Chinese' with 'Asian/Asian British'<br>• One missing case was combined with 'Preferred not to say' and 'Any other ethnic group'.		
Parent/carers' highest educational qualification	1) No qualification 2) GCSEs Grades D-G or equivalent 3) GCSEs Grades A*-C or equivalent 4) A Levels/Highers or equivalent 5) ≥Degree Level 4 NVQ/SVQ 6) 'Data unavailable' or missing.	• New category '≥Degree Level 4 NVQ/SVQ' created to account for few cases in some qualification categories, such as Masters Degree, PhD, Higher National Diploma/Certificate.<br>• 'Data unavailable' was merged with system missing		
Parent/carer mental health status	1) Never experienced a mental health problem 2) Currently or in last 6 months 3) at least 6 months ago 4) Preferred not to say or missing.	• New category 'at least 6 months ago' combined 6-18 months ago and >18 months ago to account for few cases<br>• 'Preferred not to say' was combined with system missing.		
Free School Meals (FSM) status	1) Not receiving FSM 2) Receiving FSM 3) FSM status unknown or missing	• Unknown and missing categories were combined	School records	Time1 only
Pupil Premium (PP) status	1) Not receiving PP 2) Receiving PP 3) PP status unknown or missing	• Unknown and missing categories were combined		
Special educational needs (SEN) status	1) None 2) SEN-support or equivalent in Wales and Scotland 3) Education Health and Care Plan (EHCP) or equivalent in Wales and Scotland	In line with Department for Education (DfE) definitions of SEN support and EHCPs:[2]<br>• Data from Wales: 'School action' and 'School action plus' were both recoded as 'Sen support', and 'full statement' was recoded 'EHCP' as they have similar definitions to the DfE.[3]<br>• Data from Scotland: 'Additional support needs' was recoded as 'Sen support' and 'coordinated action/support plan' as 'EHCP' as they have similar definitions to the DfE.[4]		
School stage	Primary or secondary school	• N/A - variable unchanged		
**Mental health-related moderators**				
Strengths and Difficulties Questionnaire (SDQ)[1]	Change in SDQ status between time1 and time2: 1) stays in unlikely 2) stays in possible 3) stays in probable 4) improves (from any position) 5) deteriorates (from any position).	• 25-items assess five mental health dimensions on 3-point likert scales: 1) emotional symptoms 2) conduct problems 3) hyperactivity/inattention 4) peer-relationship problems 5) pro-social behaviour (reverse-scaled).<br>• Responses are summed, higher scores indicate greater problems.<br>• A brief accompanying impact supplement describes the extent of distress, social impairment, burden and chronicity caused by a child's difficulties.<br>• We used an open-source SDQ algorithm to incorporate SDQ and impact data from multiple respondents about the same child, generating four probable disorder variables: 1)	• The SDQ and impact supplement were completed independently or in the presence of the counsellor (depending on respondent preference) by class teachers, parents/carers, and young people aged 11-19. Collected via paper questionnaire, electronic survey, or verbally reported to counsellors who record their responses	Time 1 & Time 2

		hyperactivity/inattention, 2) conduct problems, 3) emotional problems 4) all disorders (unlikely, possible, probable). The algorithm does not include the peer or social sub-scales.	(again, respondent preference).<br>• Recording as above for Time1. Time2 SDQs were not administered for pupils attending <six counselling sessions.	
School engagement and enjoyment	We calculated change in engagement/enjoyment scores between time1 and time2, and created a categorical version (reduced enjoyment/engagement, no change, improved engagement/enjoyment) to aid interpretation alongside the continuous variable.	• 6 items were measured on 3-point likert scales (0=not at all, 1=only a little, 2=quite a lot, 3=a great deal): 1) concentrates hard in lessons 2) tries to answer teachers' questions 3) enjoys classroom work 4) likes reading 5) likes writing 6) likes maths.<br>• We created a summed score due to good internal consistency of items (α=0.89),[5] inter-item correlation of r=0.44 suggesting reasonable unique variance between items[6] and face validity of item content which aligns with a multi-dimensional concept of student engagement, including behaviour, cognition, and affective elements.[7]	Routinely measured in primary school children only, using six teacher-rated items	Time 1 & Time 2
**Intervention process moderator**				
Planned ending to counselling	No<br>Yes	• We generated the planned ending variable based on whether counsellors stated counselling ended by mutual agreement or not.	Recorded by counsellors as part of a multi-response item asking them to record reason for ending counselling.	Time2
**Additional descriptive variables**				
Type of school attended	1) Academies and free schools 2) Community mainstream, community special, foundation, voluntary controlled, voluntary aided, Scotland and Wales schools 3) Other independent schools.	•	Recorded by the implementing organisation	Time1
Counselling duration	In months, based on date of first and last counselling session,	•	Recorded by counsellors	Time1 & Time2
Presenting issues (PIs)	22 possible PIs, and their severity were rated: unknown at that stage, not applicable, mild, moderate, severe.	• We recoded PIs into the following broad categories:1) internalising symptoms separation, social or general anxiety, low self-esteem, depression, mood swings, eating disorders, suicidal thoughts.[8] 2) externalising symptoms impulsivity, attentional problems, angry, emotional or callous behaviour[8] 3) peer-related problems or family tensions 4) traumatic event 5) sleeping issues 6) identity issues 7) self-destructive thoughts/acts 8) other:	Recorded by counsellors	Time1
% of counselling sessions attended	At least 1 counselling session had to have been attended for participant data to be eligible	• Attendance at planned counselling sessions was influenced by child factors (e.g. absent from school), school related factors (e.g. closures), and counsellor related (e.g. absence)	Recorded by counsellors	Time2
and % of counselling sessions missed of those offered due to child-related factors	At least 1 counselling session had to have been attended for participant data to be eligible	• Only child-related reasons for a counselling session were included in the numerator.	Recorded by counsellors	Time2

References to support Table S2

1. Goodman R. The Strengths and Difficulties Questionnaire: A Research Note. Journal of Child Psychology and Psychiatry. 1997;**38**(5):581-6.
2. Department for Education. Children with special educational needs and disabilities (SEND) [Internet]. GOV.UK. [Date accessed 24/05/2023]. Available from: https://www.gov.uk/children-with-special-educational-needs/extra-SEN-help
3. Welsh Government. The additional learning needs transformation programme: frequently asked questions 2020 [Available from: https://www.gov.wales/additional-learning-needs-transformation-programme-frequently-asked-questions-html.
4. ParentZone Scotland. Types of plan: Education.gov.scot; [Available from: https://education.gov.scot/parentzone/additional-support/how-schools-plan-support/types-of-plan/.
5. Tavakol M, Dennick R. Making sense of Cronbach's alpha. Int J Med Educ. 2011;**2**:53-5.
6. Piedmont RL. Inter-item Correlations. In: Michalos AC, editor. Encyclopedia of Quality of Life and Well-Being Research. Dordrecht: Springer Netherlands; 2014. p. 3303-4.
7. Kelders SM, van Zyl LE, Ludden GDS. The Concept and Components of Engagement in Different Domains Applied to eHealth: A Systematic Scoping Review. Front Psychol. 2020;**11**:926.
8. Woodman AC, Mailick MR, Greenberg JS. Trajectories of internalizing and externalizing symptoms among adults with autism spectrum disorders. Dev Psychopathol. 2016;**28**(2):565-81.

Table S3: Baseline mental health characteristics and presenting issues by baseline school attendance (n=7405[a] unless stated)

Characteristics		Total n (%) within sample	Median % school attendance the term before counselling (25th,75th percentiles)	n (%) Persistent absence[b] the term before counselling
**SDQ[c]-based psychiatric diagnosis prediction[d,e]**				
Hyperactivity disorder	Unlikely	3751 (51·0)	96 (91,99)	840 (22·4)
	Possible	2194 (29·8)	96 (90,99)	552 (25·2)
	Probable	1414 (19·2)	95 (91,99)	337 (23·8)
Conduct disorder	Unlikely	3018 (41·0)	96 (91,99)	673 (22·3)
	Possible	1320 (17·9)	96 (91,99)	311 (23·6)
	Probable	3019 (41·0)	96 (91,99)	744 (24·6)
Emotional disorder	Unlikely	2737 (37·2)	97 (92,99)	557 (20·4)
	Possible	2747 (37·3)	96 (91,99)	641 (23·3)
	Probable	1875 (25·5)	95 (90,98)	531 (28·3)
Any disorder	Unlikely	1039 (14·1)	97 (92,100)	207 (19·9)
	Possible	1316 (17·9)	96 (92,99)	265 (20·1)
	Probable	5004 (68·0)	96 (90,99)	1257 (25·1)
**SDQ[c] prosocial categories[f]**				
Parent reported	No identified difficulties	5631 (85·2)	96 (91,99)	1279 (22·7)
	Moderate difficulties	449 (6·8)	96 (91,99)	105 (23·4)
	Severe difficulties	527 (8·0)	96 (91,99)	126 (23·9)
Teacher reported	No identified difficulties	4242 (60·0)	96 (91,99)	958 (22·6)
	Moderate difficulties	999 (14·1)	96 (91,99)	247 (24·7)
	Severe difficulties	1833 (25·9)	96 (91,99)	451 (24·6)
Self-reported (subsample ≥11 years)	No identified difficulties	1425 (88·9)	95 (89,98)	449 (31·5)
	Moderate difficulties	109 (6·8)	94 (86,98)	43 (39·5)
	Severe difficulties	69 (4·3)	93 (86,97)	27 (39·1)
**Presenting issues: any severity** (multi-response)	Internalising symptoms[g]	7047 (95·2)	96 (91,99)	1654 (23·5)
	Externalising symptoms[h]	6270 (84·7)	96 (91,99)	1487 (23·7)
	Peer-related, bullying and/or family tension	6481 (87·5)	96 (91,99)	1538 (23·7)
	Traumatic event	3640 (49·2)	95 (90,99)	945 (26·0)
	Sleeping problem	2110 (28·5)	95 (90,98)	580 (27·5)
	Identity-related	1569 (21·2)	96 (91,99)	343 (21·9)
	Self-destructive thoughts or acts	1176 (15·9)	96 (91,99)	285 (24·2)
	Other	269 (3.6)	95 (90,98)	71 (26·4)
**Presenting issues: severe** (multi- response)	Internalising symptoms[g]	2445 (33·0)	95 (90,98)	654 (26.8)
	Externalising symptoms[h]	1882 (25·4)	95 (90,98)	502 (26·7)
	Peer-related, bullying and/or family tension	2130 (28·8)	96 (90,99)	534 (25·1)
	Traumatic event	1159 (15·7)	95 (89,99)	328 (28·3)
	Sleeping problem	335 (4·5)	94 (86,98)	130 (38·8)
	Identity-related	219 (3·0)	96 (90,98)	57 (26·0)
	Self-destructive thoughts or acts	177 (2·4)	95 (89,98)	55 (31·1)
	Other	79 (1·1)	95 (89,98)	22 (27·9)
**Total number of presenting issues (any severity)**	None recorded	124 (1·7)	96 (90,99)	35 (28·2)
	1	195 (2·6)	96 (92, 100)	45 (23·1)
	2	613 (8·3)	96 (92,100)	122 (19·9)
	3	2015 (27·2)	96 (91,99)	453 (22·5)
	4	2301 (31·1)	96 (91,99)	522 (22·7)
	5	1344 (18·2)	96 (90,99)	351 (26·1)
	6 or more	813 (11·0)	95 (90,98)	221 (27·2)
**TOTAL**	-	**7405 (100)**	**96 (91,99)**	**1749 (23·6)**

a The denominator includes data from first counselling episodes only in cases where young people had received >1 counselling episode.
b Persistent absence is defined as ≤90% school attendance of total offered school sessions, in line with the Department for Education's 2019 definition: https://assets.publishing.service.gov.uk/government/uploads/system/uploads/attachment_data/file/787314/Guide_to_absence_statistics_21032019.pdf (accessed 09/08/22)
c SDQ= Strengths and Difficulties Questionnaire
d The Youth in Mind SDQ predictive algorithm (2016) predicts four disorders using multi-informant SDQ scores. 21·7% of first counselling episodes in our sample (n=1603/7405) included self, teacher and parent SDQ data for children aged ≥11, and the remaining 78·4% included teacher and parent SDQ data only. For syntax see https://www.sdqinfo.org/py/sdqinfo/c0.py (accessed 09/08/22)
e Missing data for SDQ predicted diagnoses: 0·6% (46) hyperactivity, emotional items and 'any disorder' prediction; 0·7% (48) missing conduct items
f Missing data for prosocial SDQ items: Parent 10·8% (798) Teacher 4·5% (331), Child 66·7% (4939) largely because only a subset of children >10 years of age were eligible to complete the SDQ. Missing categories for teacher and parent prosocial variables were created to increase stability in subsequent regression models.
g Internalising symptoms included: separation, social or general anxiety, low self-esteem, depression, mood swings, eating disorders, suicidal thoughts. Presenting issues were missing for n=20/7405 (0·3%) of first counselling episodes
h Externalising symptoms included: impulsivity, attentional problems, angry, emotional or callous behaviour. Presenting issues were missing for n=20/7405 (0·3%) of first counselling episodes

Chi-Square Tests and Cramer's V to explore missing outcome data

We ran Chi square and Cramer's V tests to explore the missingness in attendance data at Time 1 and Time2 for: 1) primary vs secondary age pupils, 2) Pupil Premium (no/yes), 3) Ethnicity (6 categories), 4) SEN status (any SEN vs no SEN). We selected these variables to cover a range of different background characteristics which also had low levels of missing data in the complete sample (<1% to 9%). We ran the same analyses on country (England, Scotland, Wales) to check for potential recording differences in school attendance which could differentially affect its availability, and compared data availability at Time1 and Time2.

From 12,031 cases we excluded n= 1021 duplicate child IDs (so that the same child did not appear in the analyses more than once) and n=20 nursery age children who were not eligible for inclusion. There were 10,990 cases remaining for analyses. The results of each test are presented in Table S4.

Though there were statistically significant relationships (p<0·05) between pupil premium and SEN and school attendance data availability at one or both time points, all were weak in magnitude suggesting minimal risk of bias to the analyses based on these socio-demographic characteristics. There was no observed relationship between ethnicity or country with availability of school attendance data. Analyses of the time1 and time2 attendance data availability indicated that if children had missing data at time1 it would also be missing at time2 (true for 98%); similarly, if it was present at time1 it was usually present at time2 (true for 90%). We conclude that the missing data is related to gaps in routine data collection, and that it is not related to individual child characteristics, or due to country-level differences in the way that schools record and sharing attendance data suggesting low risk of bias to our findings based on child-level characteristics.

Table S4: Chi-Square and Cramer's V tests to assess bias in missing outcome data (n= 10,990[a] unless stated)

Time point	Characteristic						X2 (df)	P-value	Cramer's V effect size
	**Primary school**			**Secondary school**					
Time1 % missing attendance data[a]	31·80			24·45			45·2254(1)	<0·001	-0·064
Time2 % missing attendance data	36·73			34·57			3·5791(1)	0·059	-0·0180
	**SEN[b] recorded**			**No SEN recorded**					
Time1 % missing attendance data	29·04			32·14			10·3876(1)	0·001	0·0309
Time2 % missing attendance data	34·74			38·73			15·6456(1)	<0·001	0·0379
	**Pupil Premium[c]**			**No Pupil Premium**					
Time1 % missing attendance data	28·30			31·78			14·3555(1)	<0·001	0·0379
Time2 % missing attendance data	34·47			37·90			12·6625(1)	<0·001	0·0356
	**England**		**Scotland**		**Wales**				
Time1 % missing attendance data	30·27		29·39		35·25		3·5955(2)	0·166	0·0181
Time2 % missing attendance data	36·56		33·01		38·13		5·0733(2)	0·079	0·0215
	**Ethnicity**								
	**1**	**2**	**3**	**4**	**5**	**6**			
Time1 % missing attendance data	30·15	27·98	28·98	31·90	30·13	33·23	6·9292(5)	0·226	0·0251
Time2 % missing attendance data	36·30	33·93	34·14	38·06	36·66	37·24	5·7664(5)	0·330	0·0229
Time1 missing data	**Attendance data not missing Time2%**			**Attendance data missing Time2%**					
Not missing	90·96			9·04			8100(1)	<0·001	0·8595
Missing	1·05			98·95					

a Missing school attendance data were recorded as 0 (not missing) 1 (missing)
b SEN = Special Educational Needs; 85/10,990 were missing data for this variable (0·78%)
c Pupil Premium was missing for 1016/10,990 cases (9·24%)
d Ethnicity was recoded into six categories: White British=1, White Irish=2, Asian/Asian British/Chinese=3, Black/Black British=4, Mixed Ethnicity=5, Any other ethnic group=6

The association of missing SDQ data with school attendance outcomes

12·5% of SDQ data were missing , which was partly due to no Time2 SDQ data being collected for children completing fewer than six counselling sessions, or for other reasons (e.g. the child left school before Time2 SDQ data were collected). To assess potential bias from missing SDQ data in our mental health analysis, we ran a logistic regression with SDQ missing/not missing as the predictor, and attendance as the outcome. Children with missing SDQ data had 3·3-6.0% reduced attendance, and 1·9-2·6 reduced odds of persistent absence compared to those with complete SDQ data.

Table S5: Teacher-reported items about engagement and enjoyment at school, sub-sample n=4678[a,b]

Aspect of school engagement/ enjoyment	Teacher rating	Total within sample n (%)	Median % school attendance the term before counselling (25th,75th percentiles)	n (%) Persistent absence[a] the term before counselling
Concentrates hard in lessons	Not at all=0	500 (10·7)	95 (90,99)	133 (26·6)
	Only a little=1	2011 (43·1)	96 (91,99)	448 (22·3)
	Quite a lot=2	1552 (33·3)	97 (92,99)	281 (18·1)
	A great deal=3	602 (12·9)	97 (93,100)	105 (17·4)
Tries to answer teacher's questions	Not at all=0	433 (9·3)	95 (90,98)	119 (27·5)
	Only a little=1	1737 (37·2)	96 (91,99)	433 (24·9)
	Quite a lot=2	1606 (34·4)	97 (92,99)	297 (18·5)
	A great deal=3	893 (19·1)	97 (94,100)	121 (13·6)
Enjoys work	Not at all=0	345 (7·4)	95 (89,98)	101 (29·3)
	Only a little=1	1876 (40·3)	96 (91,99)	447 (23·8)
	Quite a lot=2	1846 (39·7)	97 (93,99)	328 (17·8)
	A great deal=3	587 (12·6)	97 (94,100)	91 (15·5)
Likes reading	Not at all=0	555 (11·9)	95 (90,98)	158 (28·5)
	Only a little=1	1575 (33·8)	96 (91,99)	370 (23·5)
	Quite a lot=2	1679 (36·1)	97 (93,99)	301 (17·9)
	A great deal=3	845 (18·2)	97 (93,100)	135 (16·0)
Likes writing	Not at all=0	815 (17·6)	95 (90,99)	215 (26·4)
	Only a little=1	1905 (41·0)	96 (91,99)	416 (21·8)
	Quite a lot=2	1361 (29·3)	97 (93,100)	243 (17·9)
	A great deal=3	563 (12·1)	97 (93,100)	86 (15·3)
Likes maths	Not at all=0	410 (8·9)	95 (90,99)	118 (28·8)
	Only a little=1	1779 (38·5)	96 (91,99)	417 (23·4)
	Quite a lot=2	1757 (38·0)	97 (93,99)	317 (18·0)
	A great deal=3	678 (14·7)	97 (93,100)	104 (15·3)
Sum score: median (25th, 75th percentiles)	N/A	9 (6,12)	N/A	N/A
**TOTAL**	**N/A**	**100 (4678)**	**96 (92,99)**	**970 (20·7)**

a Denominator are for first counselling episodes only, in a subsample of younger children for whom teachers reported on engagement and enjoyment items

b Missing data: concentrates hard in lessons n=13 (0·3%), tries to answer teacher's questions n=9 (0·2), enjoys work n=24 (0·5%), likes reading n=24 (0·5%), likes writing n=34 (0·7%), likes maths n=54 (1·2%); n=116 missing at least 1/6 of engagement and enjoyment items and were excluded from the summed score variable
